# Infant vitamin B12 status and its predictors – cross-sectional baseline results from an ongoing randomized controlled trial

**DOI:** 10.1016/j.ajcnut.2025.06.029

**Published:** 2025-07-01

**Authors:** Sol Maja G Bjørkevoll, Maria O'Keeffe, Carolien Konijnenberg, Beate S Solvik, Alida F Sødal, Siri Kaldenbach, Adrian McCann, Per M Ueland, Ingrid Kvestad, Elisabeth Ersvær, Mads N Holten-Andersen, Kjersti S Bakken, Tor A Strand

**Affiliations:** 1 Department of Pediatric and Adolescent Medicine, Innlandet Hospital Trust, Lillehammer, Norway; 2 Department of Global Public Health and Primary Care, Centre for International Health, University of Bergen, Bergen, Norway; 3 Department of Psychology, University of Inland Norway, Lillehammer, Norway; 4 Women’s Clinic, Innlandet Hospital Trust, Lillehammer, Norway; 5 Department of Research, Innlandet Hospital Trust, Lillehammer, Norway; 6 Bevital AS, Bergen, Norway; 7 Department of Biotechnology, University of Inland Norway, Hamar, Norway; 8 Institute of Clinical Medicine, Faculty of Medicine, University of Oslo, Oslo, Norway

**Keywords:** infants, breastfeeding, Vitamin B12, cobalamin, homocysteine, methylmalonic acid, cB12, micronutrient

## Abstract

**Background:**

Vitamin B12 is a crucial micronutrient for infant growth and development.

**Objective:**

The objective of this study was to describe vitamin B12 status in Norwegian infants aged 6–15 wk using multiple biomarkers and cut-off approaches, and to identify its predictors.

**Methods:**

From November 2021 through August 2024, infants aged 6–15 wk and their mothers were recruited from public health clinics in Innlandet County, Norway, as part of an ongoing randomized controlled trial. Plasma cobalamin and methylmalonic acid (MMA) concentrations were analyzed among all infants in the cohort (*n* = 644), and total homocysteine (tHcy) concentrations were analyzed in a subgroup (*n* = 358). The combined indicator for vitamin B12 status (cB12) was calculated by Fedosov’s equation. Low status was defined using multiple cut-off approaches. Potential predictors of infant vitamin B12 status were evaluated using regression models.

**Results:**

Mean (standard deviation [SD]) infant age was 9.1 (1.8) wk. The median (interquartile range) concentrations were: cobalamin 242 (192, 322) pmol/L, tHcy 7.4 (6.2, 9.4) μmol/L, and MMA 0.34 (0.21, 0.77) μmol/L. The mean (SD) cB12 was −0.5 (0.7). Eight percent had cobalamin <148 pmol/L, and 40% <221 pmol/L. Sixty-seven percent had tHcy >6.5 μmol/L, 19% >10 μmol/L, and 4% >13 μmol/L. Sixty-four percent had MMA>0.26 μmol/L. Exclusively breastfed infants had 40% lower cobalamin and 30% higher tHcy compared with nonbreastfed infants. Partially breastfed infants had 21% lower cobalamin and 12% higher tHcy compared with nonbreastfed infants.

**Conclusion:**

A substantial proportion of Norwegian infants have biochemical signs of low vitamin B12 status, given that the cut-offs were established in adults. Lower status was observed in partially and exclusively breastfed infants, compared with nonbreastfed infants. However, it is unclear whether these biomarker patterns have clinical significance. Further research is needed to determine consequences of low vitamin B12 biomarker concentrations in early infancy.

This trial was registered as NCT05005897

## Introduction

Vitamin B12 (cobalamin) is a water-soluble micronutrient crucial for DNA synthesis, myelination, and hematopoiesis [1]. Adequate vitamin B12 status during gestation and infancy is essential to support rapid growth and neurologic development. Severe deficiency may result in both short- and long-term consequences, including irritability, lethargy, refusal of foods, failure to thrive, developmental regression, and macrocytic anemia [2,3].

Infants are recognized as a group at risk of low vitamin B12 status [3]. However, prevalence estimates of low vitamin B12 status among infants vary widely across studies. Perhaps the main reason for this is the lack of consensus regarding the appropriate biomarkers and corresponding cut-offs for defining deficiency and low vitamin B12 status in infants. The direct biomarker, cobalamin, reflects circulating vitamin B12 status [4]. A commonly used cut-off for vitamin B12 deficiency is cobalamin <148 to 150 pmol/L, originally established in adults [4]. Studies applying this cut-off in infants have reported prevalence estimates ranging from ∼2% to >60% in different settings worldwide [[5], [6], [7], [8], [9], [10], [11], [12], [13], [14], [15], [16]].

In addition to cobalamin, functional biomarkers are often used to assess vitamin B12 status, reflecting the vitamin's role as enzymatic cofactor. Insufficient vitamin B12 availability leads to accumulation of methylmalonic acid (MMA) and total homocysteine (tHcy) [4]. A threshold of tHcy >6.5 μmol/L has been proposed for defining suboptimal vitamin B12 status in infancy, based on tHcy concentrations following cobalamin repletion [17]. Using this cut-off, >60% of infants have been classified as having suboptimal vitamin B12 status in populations with a low prevalence of vegetarian and vegan diets [12,13,17]. Recently, a lower cut-off of >5.0 μmol/L has been proposed, with a corresponding cobalamin threshold of <400 pmol/L [18]. In contrast, reference intervals (RIs) for vitamin B12 status in healthy infant populations from Canada and the United Kingdom suggest that cobalamin concentrations as low as 160–180 pmol/L may still be considered within the normal range [19,20] as well as tHcy concentrations ≥10 μmol/L [19].

The lack of agreement on the optimal biomarker and on whether RI should be defined using statistical RI from healthy infants, indicators of metabolic depletion, or thresholds associated with clinical significance, complicates the evaluation of vitamin B12 status in infancy. In this study, we investigate vitamin B12 status in a large population of presumed healthy Norwegian infants aged 6–15 wk, born to mothers with high rates of vitamin supplement use during pregnancy and lactation. We report the prevalence of low vitamin B12 status using several suggested biomarkers and cut-offs. We also explore potential predictors of vitamin B12 status to better understand the factors influencing vitamin B12 status in infancy.

## Methods

### Study design, setting, and ethics

The current report presents results from an ongoing randomized controlled trial (RCT), nested within a cohort study: vitamin B12 status in infancy and the effect of providing vitamin B12 to infants with signs of suboptimal vitamin B12 status—a registry-based, randomized controlled trial (RART). Baseline blood samples to measure vitamin B12 status were collected, and its associated factors were recorded in Norwegian infants aged 6–15 wk. In the current report, we used an observational design to examine infant vitamin B12 status and potential predictors of vitamin B12 status in the full cohort, which was 1 of 2 primary outcomes of the study [21].

**FIGURE 1:**
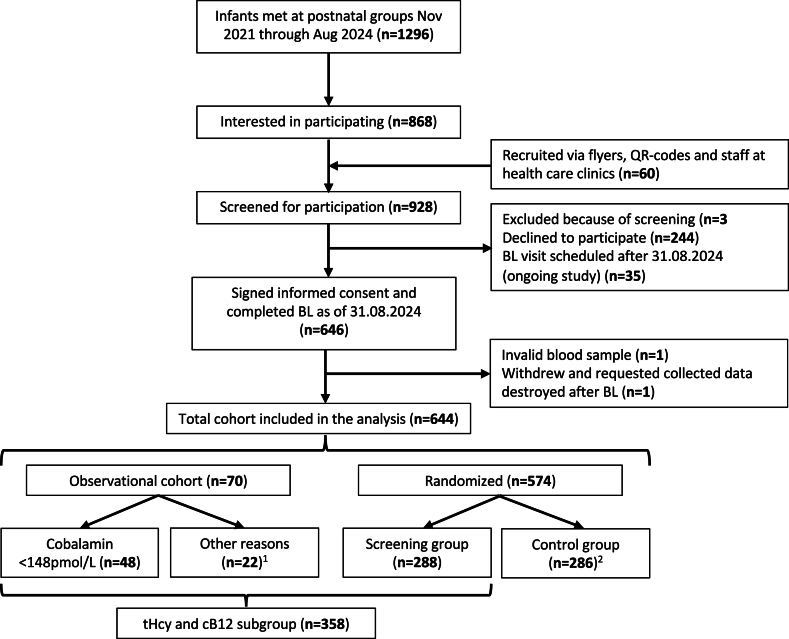
Flow chart of participants included in the registry-based, randomized controlled trial-trial and inclusion in the analysis. ^1^>13 wk *n* = 7, planning to move *n* = 3 and never randomly assigned for other reasons *n* = 11. ^2^One participant was originally randomly assigned to the control group but was later transferred to the observational cohort due to request to receive tHcy results. Abbreviations: BL, Baseline; cB12, combined indicator of B12 status; tHcy, total homocysteine.

An overview of the study is presented as a flow chart in Figure 1. Recruitment was conducted primarily by visiting postnatal groups from public healthcare clinics in 5 municipalities within Innlandet County, Norway. Eligible participants for inclusion in the study were infants with mothers who provided informed consent. Infants with severe systemic illness requiring hospitalization, growth retardation or severe congenital malformations were not included. Participants were included only in the observational part of the study, and not in the RCT, if they planned to reside outside Innlandet County the following year or if the infant was >13 wk of age at the time of the baseline assessment.

Between November 2021 and August 2024, 646 infants aged 6–15 wk and their mothers were included in the study and underwent a baseline assessment, with successful blood sampling in all but one infant. Following data collection, one participant withdrew from the study, and all collected data were destroyed. Consequently, 644 infants were included in the final analyses of the cohort, including 5 sets of twins and 12 sibling pairs. Cobalamin and MMA concentrations were measured in all infants in the cohort. Infants with plasma cobalamin concentrations <148 pmol/L were not included in the RCT and were only included in the observational part of the study. Infants with plasma cobalamin concentrations ≥148 pmol/L were included in the RCT and randomly assigned (1:1) to either a screening group or a control group. Baseline plasma samples from the screening group and observational component were analyzed for tHcy (tHcy and cb12 subgroup, *n* = 358). Infants in the control group will have their baseline plasma samples analyzed for tHcy after the 12 mo study follow-up, and are therefore not presented in the current study.

Written informed consent was obtained from those with parental responsibility of the child prior to participation. The study adhered to the principles outlined in the Declaration of Helsinki. Ethical approval was granted by the Regional Committees for Medical and Health Research Ethics South-East A (REK; ref: 186505). The trial is registered at ClinicalTrials.gov (NCT05005897).

### Data collection and analyses

#### Blood sampling

Up to 2.6 mL of blood was collected in K_2_EDTA tubes via open venous sampling from the dorsal metacarpal veins, the head (*n* = 1), or antecubital fossa (*n* = 2). If venous sampling was unsuccessful, capillary blood was obtained using a heel lance procedure (*n* = 32). A maximum of 3 attempts were made to collect a sample. To alleviate discomfort during the procedure, an oral sugar solution was provided before and during blood collection. The blood samples were centrifuged within 30 min of collection. Aliquots of the plasma fractions were either stored at 4°C for subsequent cobalamin analysis within 24 h or stored at −80°C for long-term storage. Cobalamin analysis was performed by immunoassay on the Alinity I analyzer (Abbott Laboratories) at Innlandet Hospital Trust, Lillehammer, and remaining sample was shipped to an external laboratory (Bevital AS) for the analysis of tHcy and MMA. Analysis was performed using methylchloroformate derivatization followed by quantification with GC-MS/MS [22]. The lower limit of detection (LOD) was 109.2 pmol/L for cobalamin, 0.1 μmol/L for tHcy, and 0.01 μmol/L for MMA. The upper LOD was 1475.6 pmol/L for cobalamin, 50 μmol/L for tHcy, and 10 μmol/L for MMA. For cobalamin, the within-day coefficient of variation (CV) was 12%, whereas the between-day CV was 7.2%. For tHcy and MMA, the within-day CV was 1.4% and 2.4%, respectively, and the between-day CV was 2.7% and 3.0%, respectively. Based on the performance characteristics of the 3 methods, all samples were run in singleton.

### Characteristics of mothers and their infants

Information on demographic and maternal characteristics was collected at the baseline assessment, which included an interview and a food frequency questionnaire or a 24-h dietary recall. Additional data were obtained from birth records. Maternal age was recorded in years. Prepregnancy BMI was calculated from self-reported weight before pregnancy and self-reported height, whereas BMI at baseline was derived from self-reported current weight or measured weight at the baseline assessment and self-reported height. BMI was categorized as underweight (<18.5 kg/m^2^), normal weight (18.5–24.9 kg/m^2^), overweight (25–29.9 kg/m^2^), or obese (>30 kg/m^2^). Maternal education was defined as the highest completed number of years of education and categorized as ≤ high school, ≤4 y of higher education, or >4 y of higher education. Use of vitamin B supplements during and after pregnancy was classified as yes/no, based on reported intake of B-vitamins and/or multivitamin supplements at least once per week. At the baseline assessment, mothers were also asked whether they followed a vegan or vegetarian diet (yes/no). Parity was grouped into 1, 2, or ≥3 births. Infant age at baseline was measured in weeks, and infant sex was classified as female or male. Birth weight was recorded in grams, and gestational age at delivery in weeks. Preterm delivery was defined as birth before 37 completed weeks of gestation (yes/no), and low birth weight as <2500 g (yes/no). Use of nitrous oxide (N_2_O) during labor was recorded (yes/no). Breastfeeding status was categorized as not breastfeeding, partially breastfeeding, or exclusively breastfeeding. Infant vitamin B12 supplement use (yes/no) was defined as the use of supplements containing vitamin B12 at the time of the baseline assessment.

### Cut-offs for defining vitamin B12 status

We used the following adult-derived cut-offs for cobalamin: <148 pmol/L (deficiency) and 148–221 pmol/L (low vitamin B12) [4]. We used the cut-off of >0.26 μmol/L to indicate elevated MMA [4], and cut-offs of >6.5 μmol/L [23], >10 μmol/L [24], and >13 μmol/L [4] to classify elevated tHcy. In addition to the direct and functional biomarkers, we included the combined indicator of vitamin B12 status (cB12) developed by Fedosov et al. [25]. In short, the 3 biomarker-based cB12 is cobalamin divided by the product of tHcy and MMA concentrations. Higher cB12 values indicate higher vitamin B12 status. As no validated cut-offs for cB12 exist for use in infants or children, we did not report cB12 according to any cut-offs.

### Sample size calculation

The requirement for the parent RCT defined the sample size [21]. For the present analysis, the most conservative scenario of 50% prevalence, 384 participants were needed to achieve ±5% absolute precision at 95% confidence [26]. This represents the maximum required sample size because lower or higher prevalences require fewer participants to achieve the same absolute precision.

### Statistical analysis

A statistical analysis plan was developed and approved by the authors prior to data extraction. Concentrations of cobalamin, tHcy, and MMA were expressed as medians and IQR due to their skewed distributions. Additionally, biomarker values were presented as percentages based on suggested cut-off values. The lower LOD was 109.2pmol/L for cobalamin. Values <LOD (*n* = 9, 1.4%) were replaced by univariate imputation of censored log-normal distributions, suggested by Herbers et al. [27]. None of the other biomarkers exceeded the lower or upper LOD. Before analysis, cobalamin, tHcy, and MMA values were log-transformed to approximate normal distribution. Associations between cobalamin and the other markers of vitamin B12 were estimated using Spearman’s rank-order correlation. Dose–response curves depicting the relationships between cobalamin, tHcy, and MMA, whereas adjusting for age, were made using general additive models (GAM) [28]. We used multiple generalized linear models (GLMs) with an identity link function of the Gaussian distribution family to identify predictors for the different biomarkers and cB12. Initially, predefined predictors were analyzed individually in separate GLMs. The selection of predefined predictors was based on prior knowledge and existing literature regarding factors influencing infant vitamin B12 status, ensuring the inclusion of variables that were both relevant and had sufficient sample sizes for meaningful analysis. The predefined predictors included continuous variables [birth weight (rescaled and included in the models per 100 g higher weight), infant age at baseline, and maternal age] and categorical variables (breastfeeding status, infant sex, maternal supplementation during and after pregnancy, any use of N_2_O during labor, parity, and maternal education). To identify the most appropriate model for the adjusted regression, elastic net regression was applied. This technique was chosen for its capacity to manage situations with several potential predictors, including correlated variables [29]. GLMs were then applied to the model identified as the best fit for each biomarker. All regression analyses used listwise deletion, meaning that only participants with complete data for the variables included in each model were retained. Additionally, we created a prediction plot of biomarker concentrations as a function of infant age and breastfeeding status (exclusive, partial, and not) for infants aged 6–10 wk. Statistical analyses were conducted using Stata, version 18 (STATA Corp), whereas GAM models and figures were made in R (Version 4.42; R Core Team, 2024) using the packages *mgcv*, *ggplot2*, *dplyr*, and *patchwork*. Imputed values for cobalamin were estimated using log log-normal imputation functions from the Github repository nx10/lnormimp-r, installed via the *remotes* package.

## Results

### Characteristics of participants

Baseline characteristics of mothers and infants are presented in Table 1. In this cohort, almost all mothers were omnivores and used dietary supplements containing vitamins B during pregnancy. The mean age of the infants was 9.1 wk (range: 6–15 wk), and the majority were exclusively breastfed.

**TABLE 1:** Baseline characteristics.

Characteristics	Total cohort (*n* = 644^1^)
Mothers	
Age, y	32.1(4.2)
BMI prepregnancy, kg/m^2^, %	
<18.5	19 (3.0)
18.5–24.9	371 (58)
25–29.9	137 (21)
>30	112 (18)
BMI postpartum, kg/m^2^, %	
<18.5	8 (1.3)
18.5–24.9	270 (43)
25–29.9	205 (32)
>30	153 (24)
Education, %	
≤High school	111 (17)
≤4 y of higher education	251 (39)
>4 y of higher education	282 (44)
Marital status, %	
Single	12 (1.9)
Cohabitant	424 (66)
Married	207 (32)
Vitamin B supplements^2^, %	
During pregnancy	621 (97)
After pregnancy	271 (42)
Vegans or vegetarian, %	16 (2.5)
Parity, %	
0	294 (46)
1	268 (42)
≥2	81 (12)

Infants	
Age at blood sample, wks	9.1(1.8)
Female, %	311 (48)
Birth weight, g	3553(513)
GA at time of delivery, wks	39.5(1.6)
Preterm delivery^3^, %	38 (5.9)
Use of N_2_O during labor, %	367 (61)
Breastfeeding status, %	
Not breastfed	57 (9.1)
Partially breastfed	84 (14)
Exclusively breastfed	482 (77)
B12 supplements^4^, %	4 (0.6)

Baseline characteristics are presented as mean (SD) or n (%).

Abbreviations: GA, gestational age; N_2_O, nitrous oxide; y, years.

1 *n* = 639 for prepregnancy BMI, *n* = 636 for postpartum BMI, *n* = 643 for income, education, and parity, *n* = 640 for supplement use during pregnancy, *n* = 604 for GA at delivery, *n* = 623 for breastfeeding status and infant supplement use.

2 Self-reported intake of B vitamins and/or multivitamins.

3 <37 wk of gestation.

4 Reported intake of vitamins containing vitamin B12.

### Infant vitamin B12 status

Plasma concentrations for the infants are presented in Table 2, alongside suggested cut-off values and the respective prevalence of low vitamin B12 status. Plasma cobalamin <148 pmol/L was observed in 7.5% of the infants, whereas 32% had low vitamin B12 status (cobalamin 148–221 pmol/L). Elevated MMA concentrations were observed in 64% of the infants, whereas elevated tHcy concentrations ranged from 4.2% to 67%, depending on the cut-off used.

**TABLE 2:** Vitamin B12 status among the infants according to different biomarkers and cut-offs.

Indicator of status	Values^1^	Suggested cut off and definition	*n*	Prevalence, % (95% CI)
Cobalamin (pmol/L, *n* = 644)	242 (192, 322)	<148, “Deficiency” [4] 148–221, “Low vitamin B12” [4]	48	7.5 (5.5, 9.8)
			204	32 (28, 35)
tHcy (μmol/L, *n* = 358)	7.4 (6.2, 9.4)	>6.5 [23]	241	67 (62, 72)
		>10 [24]	68	19 (15, 23)
		>13 [4]	15	4.2 (2.4, 6.8)
MMA (μmol/L, *n* = 644)	0.34 (0.21, 0.77)	>0.26 [4]	411	64 (60, 68)
cB12 (*n* = 358)	−0.5 (0.7)	NA	NA	NA

Abbreviations: cB12, combined indicator for vitamin B12 status; MMA, methylmalonic acid; tHcy, total homocysteine.

1 Biomarker-values of vitamin B12 status is reported as median(IQR), whereas Cb12 is presented as mean(SD).

**FIGURE 2:**
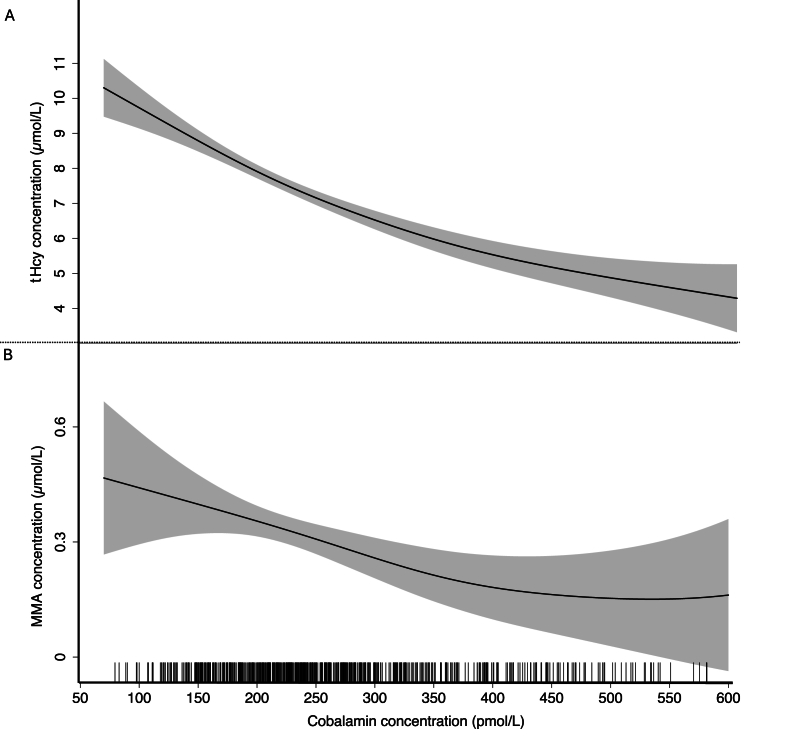
GAM plots depicting the associations between cobalamin and tHcy (panel A) and cobalamin and MMA (panel B), adjusted for infant age. Shaded areas represent 95% confidence intervals of the regression plots. Vertical lines along the x-axis represent the observed values of cobalamin concentrations in the dataset. Due to outliers, the 5% of infants with the highest MMA concentrations are not included (*n* = 32). Similarly, infants with cobalamin >600 pmol/L were not included (*n* = 11). Abbreviations: GAM, generalized additive models; MMA, methylmalonic acid; tHcy, total homocysteine.

Associations between concentrations of plasma cobalamin and the functional biomarkers, tHcy and MMA, are illustrated in Figure 2A, B. Plasma cobalamin was inversely correlated with tHcy (rho = −0.50, *P* < 0.001) and MMA (rho = −0.11, *P* = 0.005).

### Predictors of infant vitamin B12 status

**FIGURE 3:**
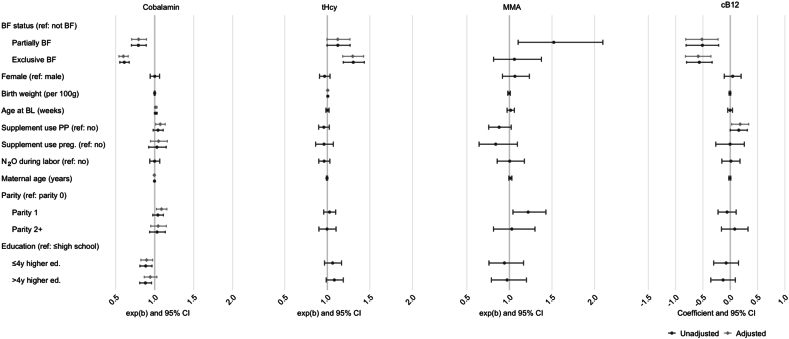
Forest plot showing unadjusted and adjusted general linear model regressions for potential predictors of infant vitamin B12 status. Adjusted models were chosen based on elastic net regression (no predictors were selected for MMA). Cobalamin, tHcy, and MMA were log-transformed, and the graphs show the exponentials of the regression coefficients, representing the relative change compared with the reference group. cB12 was not log-transformed, and the regression coefficients are displayed. Birth weight was rescaled and included in the models per 100 g higher weight. Mothers' supplement use was defined as intake of multivitamins and/or B-vitamins ≥1 d per week during pregnancy or postpartum (at the time of the BL assessment). Abbreviations: BF, breastfed; BL, baseline; cB12, combined indicator for vitamin B12 status; ed, education; MMA, methylmalonic acid; N_2_O, nitrous oxide; PP, postpartum; preg, pregnancy; tHcy, total homocysteine; y, years; wt, weight.

Predictors of infant vitamin B12 status are illustrated in Figure 3, with additional details in Supplemental Tables 1–4. Breastfeeding status was consistently associated with infant vitamin B12 status, with exclusively and partially breastfed infants showing lower vitamin B12 status compared with nonbreastfed infants. Partially breastfed infants had 21% lower cobalamin and 12% higher tHcy concentrations, whereas exclusively breastfed infants had 40% lower cobalamin and 30% higher tHcy concentrations compared with nonbreastfed infants in the adjusted models. In contrast, none of the other included potential predictors showed a consistent association with infant vitamin B12 status.

**FIGURE 4:**
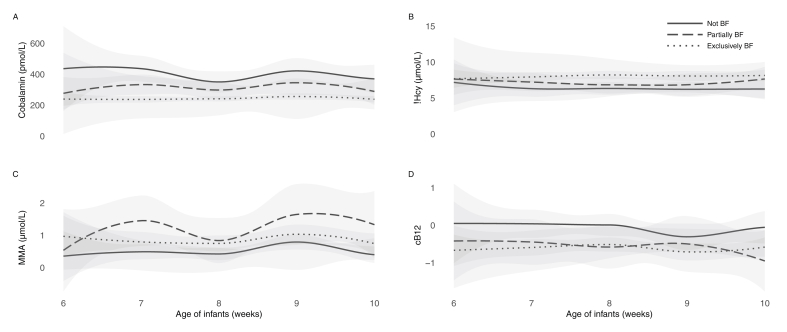
LOESS regression plots depicting the relationship between infant vitamin B12 status, age in weeks, and breastfeeding status. Panels show cobalamin (A), tHcy; (B), MMA; (C), and cB12 (D). Infants above 10 wk of age are not depicted due to few observations in each BF category. Abbreviations: LOESS, Locally Estimated Scatterplot Smoothing; BF, breastfed; cB12, combined indicator for vitamin B12 status; MMA, methylmalonic acid; tHcy, total homocysteine.

Figure 4 illustrates the relationship between infant age, breastfeeding status, and vitamin B12 markers. Plasma cobalamin concentrations were consistently lower in exclusively breastfed infants compared with partially or nonbreastfed infants, regardless of age. Similarly, the functional biomarkers tHcy and MMA were elevated among exclusively breastfed infants across all age groups**.** Supplemental Table 5 provides stratified analyses of vitamin B12 status according to breastfeeding status.

## Discussion

To our knowledge, this study is one of the largest population-based studies on infant vitamin B12 status and its predictors conducted in a high-income setting. Using adult-derived cut-offs, also commonly used in pediatric populations, we found that 40% of the infants had vitamin B12 deficiency or low vitamin B12 status (cobalamin <221 pmol/L). However, the clinical relevance of these thresholds in infancy remains uncertain. Breastfeeding practice was the strongest determinant of infant vitamin B12 status.

The observed prevalence of vitamin B12 deficiency (7.5% with cobalamin <148 pmol/L) is consistent with previous findings from Norway [[12], [13], [14]], Canada [16], and Germany [15], where reported deficiency rates among infants ranged from 2 to 15% using similar cut-offs. Higher prevalence rates have mainly been reported in populations with limited dietary diversity and low availability of vitamin B12 rich foods [[5], [6], [7], [8], [9],^11^].

The proportion of infants classified with elevated tHcy varied widely depending on the cut-off used (ranging from 4.2% with tHcy >13 μmol/L to 67% with tHcy >6.5 μmol/L). The threshold of tHcy > 6.5 μmol/L is based on observed reductions in tHcy concentrations following a high-dose cobalamin injection in healthy infants [17]. However, it remains uncertain whether exceeding this threshold has clinical consequences. This is illustrated by Ljungblad et al. [14] who reported that 78% of infants with tHcy >8 μmol/L showed no associated symptoms of vitamin B12 deficiency. As Green [30] highlighted, improvements in laboratory values alone do not necessarily indicate clinical benefit.

Although tHcy is also influenced by folate status, deficiency of this vitamin B is rarely observed in similar populations [12,13]. Therefore, the tHcy concentrations observed in this study likely reflect vitamin B12 status rather than folate deficiency. The functional marker MMA is not influenced by folate status and is therefore considered a more specific indicator of vitamin B12 status in many populations [4]. However, the relatively weak association between MMA and breastfeeding status observed in this study may reflect the influence of additional factors beyond vitamin B12 on MMA concentrations. In early life, organ immaturity and microbial activity impacting production of precursors of MMA may influence circulating concentrations [31,32]. Consequently, MMA may be a less reliable marker of vitamin B12 status in young infants, particularly in the first months of life [32].

Of the potential predictors included in our models, breastfeeding status was the strongest predictor of infant vitamin B12 status, with exclusively and partially breastfed infants having lower B12 status compared with nonbreastfed infants, similar to findings from previous studies [12,13,[33], [34], [35], [36], [37], [38], [39], [40]]. A clear gradient was observed from nonbreastfed to exclusively breastfed infants. This finding suggests that breast milk alone may provide insufficient vitamin B12. Alternatively, it is possible that formula-fed infants receive excess of vitamin B12, potentially inflating status markers.

Among infant characteristics, neither birth weight, sex, nor age at baseline showed an association with infant vitamin B12 status. Previous studies have shown that vitamin B12 status declines during the first months of life before increasing later in infancy and childhood [7,41]. In our study, we did not observe an association between age and B12 status. One possible explanation is that the narrow age range (6–15 wk) in our study limited our ability to detect an age-related association. Regarding maternal characteristics, maternal age, education, and parity did not consistently predict infant vitamin B12 status. Additionally, despite concerns that N_2_O can disrupt cobalamin metabolism and therefore be a risk factor for infant vitamin B12 deficiency [42,43], our findings suggest that N_2_O exposure during labor does not have a measurable effect on infant vitamin B12 status at 6–15 wk of age. Importantly, we did not account for the extent of N_2_O used during labor.

This study included healthy infants aged 6–15 wk, recruited through public healthcare clinics. The findings are likely generalizable to other healthy infants in Norway and similar populations. Key strengths include the large sample size and the use of multiple biomarkers to assess vitamin B12 status.

Limitations of the study include the fact that tHcy concentrations were only available for a subset of infants (i.e., the tHcy/cB12 subgroup), which does not include the control group from the RCT. This means that the actual tHcy concentration in the full sample is likely slightly lower. However, this difference is minimal, as the median tHcy concentration was 7.2 μmol/L in the screening group and 7.4 μmol/L in the full sample. Another limitation is the reliance on single blood samples collected at baseline. Although repeated measurements would have been beneficial to account for intraindividual variation and minimize the impact of sampling and analytical errors, this was not feasible due to ethical and logical constraints in infant research. Further, the intraclass correlation coefficients for the 3 biomarkers of interest have previously been reported to be high (≥0.75) [44], suggesting that their measurement at a single timepoint is likely reliable, accurate, and reproducible. Finally, 5%–10% of the samples were capillary rather than venous, which may influence biomarker concentrations due to red cell lysis. However, we found no correlations between biomarker concentrations and the hemolysis index (data not shown).

We observed a high proportion of infants with biochemical signs of low vitamin B12 status, even in this favorable setting of healthy infants whose mothers reported high use of supplements. This raises the question of whether these biomarker concentrations reflect true deficiency or normal variation in infancy. Although early identification and treatment of clinically relevant deficiency are important to prevent irreversible consequences, unnecessary treatment should be avoided. There is an urgent need for studies with clinical endpoints to establish which biomarker levels truly indicate deficiency in infants. Ultimately, the goal must be to balance timely identification and treatment of at-risk infants while avoiding overtreatment in those with normal status.

## Author contributions

The authors’ responsibilities were as follows – TAS, KSB, IK, CK: conceived the study; SMGB, MOK, CK, BSS, AFS, SK, EE, MNHA, KSB, TAS: acquired the data; SMGB, CK, TAS, IK, KSB: developed the analysis plan for the manuscript. AM, PMU: analyzed blood samples, SMGB, MOK: analyzed the data, performed the statistical analysis, and wrote the first draft of the manuscript; SMGB, MOK, CK, TAS, KSB, MNHA, EE, BSS: contributed to multiple rounds of manuscript revisions, while all authors contributed to the critical review of the final version and all authors: read and approved the final manuscript.

## Data availability

Data described in the manuscript, code book, and analytic code will be made available upon request pending applications sent to the authors or by contacting Innlandet Hospital Trust at ihop@sykehuset-innlandet.no. To meet ethical requirements for the use of patient data, requests must be approved by the Regional Committee for Medical and Health Research Ethics in Norway and the Innlandet Hospital Trust.

## Funding

This work was supported by South-Eastern Norway Regional Health Authority (grant no. 2020096) (to TAS), by Norwegian Regional Research Fund (grant no. 332775) (to KSB) and by Innlandet Hospital Trust (grant no. 150455 and grant no. 150473) (to TAS).

## Conflict of interest

The authors report no conflicts of interest.
