# Supplementary material for: Infant vitamin B12 status and its predictors – cross-sectional baseline results from an ongoing randomized controlled trial
Source: Am J Clin Nutr. 2025 Jul 1;122(3):803–10. doi: 10.1016/j.ajcnut.2025.06.029 (PMC12489377; doi:10.1016/j.ajcnut.2025.06.029)
Supplement: multimedia component 1 [file mmc1.docx]

Infant Vitamin B12 Status and its Predictors – Cross-Sectional Baseline Results from an Ongoing Randomized Controlled Trial

First author: Sol Maja G Bjørkevoll

**Supplementary table 1: Generalized linear models of cobalamin and potential predictors**

| Unadjusted models | Predictor | Exp(b) | Lower CI | Upper CI |
| --- | --- | --- | --- | --- |
| Infant characteristics | BF-status (not BF = ref.) |  |  |  |
|  | Partially BF | 0.791 | 0.702 | 0.892 |
|  | Exclusively BF | 0.610 | 0.553 | 0.673 |
|  | Female | 1.000 | 0.942 | 1.063 |
|  | Birth wt, per 100g | 0.999 | 0.994 | 1.005 |
|  | Age at BL, weeks | 1.013 | 0.996 | 1.031 |
| Maternal characteristics | Supplement preg. (no = ref.) | 1.029 | 0.923 | 1.147 |
|  | Supplement PP (no = ref.) | 1.042 | 0.980 | 1.108 |
|  | N_2_O-use (no = ref.) | 0.999 | 0.937 | 1.065 |
|  | Maternal age, y | 0.997 | 0.990 | 1.004 |
|  | Parity (parity 0 = ref.) |  |  |  |
|  | Parity 1 | 1.041 | 0.975 | 1.111 |
|  | Parity 2+ | 1.030 | 0.936 | 1.135 |
|  | Education ($\leq$High school = ref.) |  |  |  |
|  | $\leq$4y higher ed. | 0.885 | 0.811 | 0.965 |
|  | >4y higher ed. | 0.880 | 0.808 | 0.959 |
| Adjusted model | Predictor | Exp(b) | Lower CI | Upper CI |
| Infant characteristics | BF-status (Not BF = ref.) |  |  |  |
|  | Partially BF | 0.793 | 0.704 | 0.894 |
|  | Exclusively BF | 0.598 | 0.541 | 0.660 |
|  | Age at BL, weeks | 1.014 | 0.998 | 1.030 |
| Maternal characteristics | Supplement preg. (no = ref.) | 1.049 | 0.947 | 1.162 |
|  | Supplement PP (no = ref.) | 1.071 | 1.011 | 1.135 |
|  | Maternal age, y | 0.995 | 0.988 | 1.003 |
|  | Parity (parity 0 = ref) |  |  |  |
|  | Parity 1 | 1.087 | 1.022 | 1.156 |
|  | Parity 2+ | 1.044 | 0.949 | 1.149 |
|  | Education ($\leq$High school = ref.) |  |  |  |
|  | $\leq$4y higher ed. | 0.895 | 0.823 | 0.974 |
|  | >4y higher ed. | 0.943 | 0.866 | 1.027 |

Unadjusted GLMs were performed where each potential predictor was analyzed individually against cobalamin. Adjusted GLMs were conducted based on the results from Elastic Net regression. The selected predictors were then entered into a GLM. Cobalamin were log-transformed, and exponentials of regression coefficients (and confidence intervals) show in the table, these vales represents the relative change compared to the reference group, i.e. the cobalamin concentration is 21% lower among partially BF infants compared to not BF infants. Maternal supplement use was defined as intake of multivitamins and or B-vitamins more than 1 day per week during pregnancy or postpartum (at the time of the baseline assessment). Abbreviations: BF, breastfed; BL, baseline; ed., education; GLM, generalized linear model; N_2_O, nitrous oxide; PP, postpartum; preg., pregnancy; y, years; wt, weight.

**Supplementary table 2: Generalized linear models of tHcy and potential predictors**

| Unadjusted models | Predictor | Exp(b) | Lower CI | Upper CI |
| --- | --- | --- | --- | --- |
| Infant characteristics | BF-status (not BF = ref.) |  |  |  |
|  | Partially BF | 1.124 | 0.996 | 1.267 |
|  | Exclusively BF | 1.305 | 1.187 | 1.434 |
|  | Female | 0.968 | 0.908 | 1.032 |
|  | Birth wt, per 100g | 1.006 | 1.000 | 1.012 |
|  | Age at BL, weeks | 1.002 | 0.984 | 1.020 |
| Maternal characteristics | Supplement preg. (no = ref.) | 0.961 | 0.863 | 1.070 |
|  | Supplement PP (no = ref.) | 0.958 | 0.898 | 1.023 |
|  | N_2_O-use (no = ref.) | 0.963 | 0.900 | 1.030 |
|  | Maternal age, y | 0.996 | 0.989 | 1.004 |
|  | Parity (parity 0 = ref.) |  |  |  |
|  | Parity 1 | 1.026 | 0.958 | 1.098 |
|  | Parity 2+ | 0.997 | 0.901 | 1.102 |
|  | Education ($\leq$High school = ref.) |  |  |  |
|  | $\leq$4y higher ed. | 1.063 | 0.967 | 1.167 |
|  | >4y higher ed. | 1.082 | 0.987 | 1.187 |
| Adjusted model | Predictor | Exp(b) | Lower CI | Upper CI |
| Infant characteristics | BF-status (Not BF = ref.) |  |  |  |
|  | Partially BF | 1.122 | 0.995 | 1.266 |
|  | Exclusively BF | 1.298 | 1.181 | 1.427 |
|  | Birth wt, per 100g | 1.004 | 0.999 | 1.010 |

Unadjusted GLMs were performed where each potential predictor was analyzed individually against t-Hcy. Adjusted GLMs were conducted based on the results from Elastic Net regression. The selected predictors were then entered into a GLM. tHcy were log-transformed, and exponentials of regression coefficients (and confidence intervals) show in the table, these vales represents the relative change compared to the reference group, i.e. the tHcy concentration is 12.4% higher among partially BF infants compared to not BF infants. Maternal supplement use was defined as intake of multivitamins and or B-vitamins more than 1 day per week during pregnancy or postpartum (at the time of the baseline assessment). Abbreviations: BF, breastfed; BL, baseline; ed., education; GLM, generalized linear model; N_2_O, nitrous oxide; PP, postpartum; preg., pregnancy; y, years; wt, weight; tHcy, total homocysteine.

**Supplementary table 3: Generalized linear models of MMA and potential predictors**

| Unadjusted models | Predictor | Exp(b) | Lower CI | Upper CI |
| --- | --- | --- | --- | --- |
| Infant characteristics | BF-status (not BF = ref.) |  |  |  |
|  | Partially BF | 1.521 | 1.103 | 2.098 |
|  | Exclusively BF | 1.058 | 0.814 | 1.376 |
|  | Female | 1.065 | 0.918 | 1.234 |
|  | Birth wt, per 100g | 0.995 | 0.981 | 1.010 |
|  | Age at BL, weeks | 1.014 | 0.973 | 1.058 |
| Maternal characteristics | Supplement preg. (no = ref.) | 0.839 | 0.644 | 1.094 |
|  | Supplement PP (no = ref.) | 0.879 | 0.757 | 1.021 |
|  | N_2_O-use (no = ref.) | 1.003 | 0.856 | 1.176 |
|  | Maternal age, y | 1.011 | 0.993 | 1.029 |
|  | Parity (parity 0 = ref.) |  |  |  |
|  | Parity 1 | 1.220 | 1.041 | 1.429 |
|  | Parity 2+ | 1.028 | 0.813 | 1.300 |
|  | Education ($\leq$High school = ref.) |  |  |  |
|  | $\leq$4y higher ed. | 0.941 | 0.760 | 1.166 |
|  | >4y higher ed. | 0.972 | 0.788 | 1.200 |

Unadjusted GLMs were performed where each potential predictor was analyzed individually against MMA. Elastic Net regression did not identify any predictors for the adjusted model. MMA were log-transformed, with regression coefficients representing the relative change compared to the reference group. Maternal supplement use was defined as intake of multivitamins and or B-vitamins more than 1 day per week during pregnancy or postpartum (at the time of the baseline assessment). Abbreviations: BF, breastfed; BL, baseline; ed., education; GLM, generalized linear model; MMA, methylmalonic acid; N_2_O, nitrous oxide; PP, postpartum; preg., pregnancy; y, years; wt, weight.

**Supplementary table 4: Generalized linear models of cB12 and potential predictors**

| Unadjusted models | Predictor | Exp(b) | Lower CI | Upper CI |
| --- | --- | --- | --- | --- |
| Infant characteristics | BF-status (not BF = ref.) |  |  |  |
|  | Partially BF | -0.510 | -0.808 | -0.212 |
|  | Exclusively BF | -0.567 | -0.801 | -0.332 |
|  | Female | 0.044 | -0.110 | 0.198 |
|  | Birth wt, per 100g | -0.006 | -0.020 | 0.008 |
|  | Age at BL, weeks | -0.003 | -0.046 | 0.041 |
| Maternal characteristics | Supplement preg. (no = ref.) | -0.004 | -0.265 | 0.257 |
|  | Supplement PP (no = ref.) | 0.156 | -0.000 | 0.312 |
|  | N_2_O-use (no = ref.) | 0.012 | -0.154 | 0.179 |
|  | Maternal age, y | -0.007 | -0.025 | 0.010 |
|  | Parity (parity 0 = ref.) |  |  |  |
|  | Parity 1 | -0.058 | -0.224 | 0.108 |
|  | Parity 2+ | 0.082 | -0.161 | 0.325 |
|  | Education ($\leq$High school = ref.) |  |  |  |
|  | $\leq$4y higher ed. | -0.076 | -0.304 | 0.151 |
|  | >4y higher ed. | -0.132 | -0.356 | 0.092 |
| Adjusted model | Predictor | Exp(b) | Lower CI | Upper CI |
| Infant characteristics | BF-status (Not BF = ref.) |  |  |  |
|  | Partially BF | -0.519 | -0.815 | -0.223 |
|  | Exclusively BF | -0.587 | -0.820 | -0.354 |
| Maternal characteristics | Supplement PP (no = ref.) | 0.182 | 0.026 | 0.338 |

Unadjusted GLMs were performed where each potential predictor was analyzed individually against cB12. Adjusted GLMs were conducted based on the results from Elastic Net regression. The selected predictors were then entered into a GLM. Maternal supplement use was defined as intake of multivitamins and or B-vitamins more than 1 day per week during pregnancy or postpartum (at the time of the baseline assessment). Abbreviations: BF, breastfed; BL, baseline; cB12, combined indicator for vitamin B12 status; ed., education; GLM, generalized linear model; N_2_O, nitrous oxide; PP, postpartum; preg., pregnancy; y, years; wt, weight.

**Supplementary table 5: Vitamin B12 status according to breastfeeding status**

|  | Exclusive BF | Partially BF | Not BF |
| --- | --- | --- | --- |
| Cobalamin (pmol/L) | 229 (185, 283) | 272 (215, 425) | 379 (302, 446) |
| tHcy (µmol /L) | 7.9 (6.5, 9.7) | 6.6 (6.1, 8.1) | 5.9 (5.3, 7.0) |
| MMA (µmol/L) | 0.3 (0.2, 0.7) | 0.5 (0.3, 1.3) | 0.4 (0.2 (0.6) |
| cB12 | -0.5, (-1.0, -0.1) | -0.5 (-0.8, 0.0) | 0.0 (-0.3, 0.4) |

The figure displays the median (IQR) biomarker and cB12 values according to BF-status. for cobalamin and MMA; Exclusively BF n= 482, partially BF n=84 and not BF n=57. For tHcy and cB12; Exclusively BF n= 255, partially BF n=49 and not BF n=44. Abbreviations: cB12, combined indicator for vitamin B12 status, MMA, methylmalonic acid; tHcy, total homocysteine; BF, breastfed.
